# Factors associated with non-utilization of long acting and permanent contraceptive methods among married women of reproductive age in Chencha district, Southern Ethiopia: a case-control study

**DOI:** 10.11604/pamj.2020.35.109.20683

**Published:** 2020-04-08

**Authors:** Andamlak Gizaw Alamdo, Mesfin Kote Debere, Zemedu Mehamed Tirfe

**Affiliations:** 1 Department of Public Health, Saint Paul's Hospital Millennium Medical College, Addis Ababa, Ethiopia; 2 College of Medicine and Health Sciences, Arba Minch University, Arba Minch, Ethiopia; 3 Ethiopian Public Health Institute, Ministry of Health, Addis Ababa, Ethiopia

**Keywords:** Non-utilization, married women, reproductive age, Ethiopia

## Abstract

**Introduction:**

In many developing countries like Ethiopia, access and the utilization of long acting and permanent contraceptive methods (LAPCMs) is very low and it is also difficult to find them from many reproductive health programs. The aim of this study was to assess factors associated with non-utilization of long-acting and permanent contraceptive methods among married women in the reproductive age (15-49 years).

**Methods:**

A community based unmatched case-control study was conducted in Chencha district, Southern Ethiopia from November 2015 to December 2015. Cases were those women who used contraceptive methods other than LAPCMs and women who were not using contraceptive methods. Controls were women who used LAPCMs in their lifetime. Study participants were selected by simple random sampling technique and records were reviewed and then by tracing their address, the selected samples were interviewed. We analyzed data using SPSS version 20.0 and logistic regression models to identify associated factors.

**Results:**

We enrolled 328 women: factors such as partner’s lower level of education (p = 0.003), less number of live children (p = 0.04), preference to have children in the future (p = 0.042), husband’s approval to LAPCMs (p = 0.002), not informed to use contraception (p = 0.006), started using contraceptives during campaign (p = 0.021) and discussion with health professionals (p = 0.039) were predictors of non-utilization of LAPCMs.

**Conclusion:**

Non-utilization of LAPCMs in the district is associated with knowledge about LAPCMs and quality of health service. Interventions should focus on couple’s knowledge, training of service providers in quality care, and the rights of clients, informed choice to contraceptive methods.

## Introduction

Most maternal morbidity and mortality in the developing world are the result of unintended pregnancy [1,2] and more than half of all pregnancies are unintended or mistimed [1]. Globally, the total number of maternal deaths dropped from 523 000 in 1990 to 358 000 in 2008. About 99% (355,000) of the maternal deaths occurred in the developing countries. Of which, 87% (313,000) of these deaths occurred in sub-Saharan Africa and South Asia [1]. High rates of infant, child and maternal mortality can be averted by using modern contraceptive methods [1,3]. Access to contraceptive methods, particularly long acting and permanent contraceptive methods (LAPCMs) would benefit women to have a safe pregnancy and protect them from complications of pregnancy [4]. Long-acting and permanent contraceptives are better contraceptives based on effectiveness, length of effectiveness, reversibility, the importance of a rapid and predictable return of fertility after stopping a method if the user decides to get pregnant [3]. However, LAPCMs are not readily available for use in most of the developing world and also LAPCMs are not included in many of the reproductive health programs of these counties [4]. Ethiopia has a total fertility rate (TFR) of 4.6 children per women and has a high unmet need for family planning (22%). One of the strategies to avert such problems is improving the accessibility of LAPCMs [2,5]. Most of the currently married women in Ethiopia are utilizing short-term contraceptive methods like injectables and pills [2,6,7]. Injectables account for 23% of the total contraceptive use, followed by implants (8%), and intrauterine contraceptive device (IUCD) (2%) [5].

Barriers to the use of LAPCMs are multifactorial and exist at the patient, provider, health system, and national levels [6]. Risk factors such as negative attitude to the methods, educational level, and knowledge on LAPCMs and perception on partner's support of LAPCMs use have been mentioned as the reasons not to use LAPCMs in Ethiopia [8,9]. Information on factors associated with the very low use of LAPCMs using strong study design is crucial for meeting the needs of women and ensuring safe and effective protection from unintended pregnancy and also to prevent maternal and newborn deaths [2,5,10]. Chencha district, which is found in Southern Ethiopia was one among some districts having a high unmet need for family planning for married women age 15-49 in the region and there was also high birth rate in the area [5]. The aim of this study was to assess factors associated with utilization of long-acting contraceptive methods among married women of reproductive age in Chencha district, Southern Ethiopia.

## Methods

**Study area and settings:** a community based unmatched case-control study was conducted from November 2015 to December 2015 in Chencha district, Gamo-Gofa zone, in Southern Ethiopia. According to the 2007 census result, the district has a population of 140,183 and of this 68,970 were males and 71, 213 were females [11]. The district is divided into 50 kebeles (the smallest current governmental administrative unit). Forty-five of them are rural and the rest 5 are urban kebeles.

**Sampling:** we determined a sample size of 332 currently married women aged 15-49 years using a two population proportion formula. We considered a similar study conducted in Tigray region in Northern Ethiopia and used women who had less than two pregnancies as the main predictor of not utilizing LAPCMs [7]. The prevalence of women who had less than two pregnancies was 59.9% for women who were not utilizing LAPCMs and we assumed 95% confidence level, 80% power and case to control ratio was 1:1 for 151 cases and 151 controls, and adding 10% non-response rate. A total of 328 participants were finally recruited for the study. Out of the district's 50 kebeles, we selected 13 randomly. First, we reviewed and then listed women of reproductive age from registration books in each kebele. Women of reproductive age were divided into women who were using contraceptive methods other than LAPCMs and women who were not using contraceptive methods and categorized as cases. Those women who have been using LAPCMs during or any time before were taken to be controls. Study participants were selected by simple random sampling with proportional allocation to the population size.

**Data collection:** using a structured questionnaire, which was adapted from similar studies, the data collectors interviewed the study participants. Questionnaires were first prepared in English then translated into the official language of the Southern region (Amharic) by a language expert. To check whether the translation was consistent with the English version, the questionnaire was back-translated into English by another language expert. Six trained health science diploma holders who were fluent in the local language and two supervisors were recruited to collect data. The data collectors were responsible for arranging the respondents, giving clarifications, reviewing records and interviewing the respondents and assisting in any difficulties they have during an interview of the questionnaires. The supervisors were responsible for leading the whole data collection process, to check the data for consistency, completeness and any irregularities. Correction measures were taken in the field on daily basis. To minimize the non-response rate, we visited the houses repeatedly.

**Data processing and analysis:** the data was entered into EPI info version 3.5.1 and it was transferred into SPSS version 20.0 software for analysis. First, descriptive analyses were carried out for each of the variables. Unconditional logistic regression analysis was performed to examine the effect of each variable of interest in the low utilization of long-acting and permanent contraceptive methods. Hosmer and Lemeshow goodness of fit was used to see the model fit for the variables. Bivariate analysis was done to see the association between the explanatory and outcome variables. To control the effect of possible confounders', variables found to be potential factors for low utilization of long-acting and permanent contraceptive methods was taken and included in the unconditional logistic regression model. A logistic regression model was used to determine odds ratio (OR) and 95% confidence interval (CI) for the different factors of non-utilization of LAPCMs. In the model, a backward stepwise analysis was employed. All variables were treated as categorical.

**Ethical considerations:** ethical clearances for the proposed study were obtained from Arba Minch College of Health Sciences Institutional Research Ethics Review Committee. Permission from Chencha district health office was obtained. Since some of the interview questions involved on study participants private life, a written and verbal consent from all the participants was obtained and all participants signed on a consent form. The ethics committee that approved this study also approved the mode of consent. Confidentiality of the information was maintained throughout the study by excluding names as identification in the data. We assured all the information gathered during the course of the study was kept completely confidential. All the information that was delivered to us was coded for anonymity. Only the investigators have access to the data.

## Results

**Sociodemographic and socioeconomic characteristics of married women:** we interviewed 328 currently married women with a respondent rate of 98.7%. The median age of the respondents was 30 years (29 years for cases and 30 years for controls) and the inter-quartile range was 6 and 7 for cases and controls. Geographically, 133(81.1%) cases and 142(86.6%) controls were from rural areas. Among the respondents, 98(59.8%) cases and 101(61.6%) controls were Orthodox by religion and 66(40.2%) cases and 61(37.2%) controls were Protestant. Among the respondents, 48(29.3%) cases and 58(34.5%) controls can't read and write. Concerning occupation of the respondents, 50(30.5%) cases and 60(36.6%) controls were housewife and 56(34.1%) cases and 52(31.7%) controls were merchant (Table 1).

**Table 1:** Socio-demographic factors associated with married women’s non-utilization of LAPCMs in bivariable analysis, Chencha district, Southern Ethiopia, 2016

Variables		Cases (n=164)	Controls (n=164)	COR(95%CI)	p-value
**Age**	15-24	17(10.4%)	16(9.8%)	2.80(1.05-7.41)	0.038*
	25-29	71(43.3%)	47(28.7%)	3.98(1.81-8.73)	0.001*
	30-34	40(24.4%)	35(21.3%)	3.01(1.31-6.90)	0.009*
	35-39	25(15.2%)	37(22.6%)	1.78(.75-4.20)	
	40-49	11(6.7%)	29(17.7%)	1.00	0.188
**Educational level of respondent**	Can’t read and write	48(29.3%)	58(35.4%)	1.15(.53-2.49)	0.706
	Primary school	54(32.9%)	49(29.9%)	1.54(.71-3.32)	0.268
	Secondary& preparatory	47(28.7%)	36(22%)	1.82(.82-4.03)	0.136
	College & University	15(9.1%)	21(12.8%)	1.00	
**Educational level of partner**	Can’t read and write	31(18.9%)	39(23.8%)	1.49(.76-2.94)	0.245
	Primary school	62(37.8%)	38(23.2%)	3.06(1.63-5.76)	0.001*
	Secondary& preparatory	46(28%)	40(24.4%)	2.16(1.13-4.11)	0.019*
	College & University	25(7.6%)	47(28.7%)	1.00	
**Religion**	Orthodox	98(59.8%)	101(61.6%)	0.89(.57-1.40)	0.632
	Protestant	66(40.2%)	61(37.2%)	0	0.999
	others	0	2(1.2%)	1.00	
**Monthly income**	Less than 200	31(18.9%)	21(12.8%)	1.91(.82-4.43)	0.131
	200 up to 500	78(47.6%)	66(40.2%)	1.52(.75-3.11)	0.243
	500 up to 1000	22(13.4%)	31(18.9%)	0.91(.39-2.11)	0.842
	1000 up to 2000	16(9.8%)	24(14.6%)	0.86(.35-2.11)	0.746
	Greater than 2000	17(10.4%)	22(13.4%)	1.00	

**Reproductive health history of married women:** the median age at marriage for cases was 21 years and for controls was 20 years. The median age at first birth was 21 for cases and 22 years for controls. The majority of cases (48.2%) and controls (61.0%) had more than two children born alive and around 20(12.2%) cases and 28(17.1%) of controls face abortion in their lifetime. Thirty-seven (22.6%) of cases and 38(67.7%) of controls had lost children in their lifetime. Over 86% of cases (86.6%) and 111(33.8%) of controls noted they planned to have children in the future. The majority 93(56.7%) cases and 78(47.6%) controls preferred to have children after two years. One hundred forty-two (86.6%) of cases' and 117(71.3%) of controls' husband want children in the future (Table 2).

**Table 2:** Reproductive health history related factors associated with married women’s non-utilization of LAPCMs in bivariable analysis, Chencha district, Southern Ethiopia, 2016

Variables		Cases (n=164)	Controls (n=164)	COR(95%CI)	p-value
**Number of live children**	≤ 4	133(81.1%)	114(69.5%)	0.82(.73-.92)	0.001*
	>4	27(16.5%)	50(30.5%)	1.00	
**Like to have children in the future**	Yes	142(86.6%)	111(67.7%)	3.08(1.76-5.37)	0.001*
	No	22(13.4%)	53(32.3%)	1.00	
**Number of children in the future**	1	48(29.3%)	32(19.5%)	1.45(.75-2.81)	0.266
	2	60(36.6%)	48(29.3%)	1.21(.65-2.24)	0.541
	>2	33(20.1%)	32(19.5%)	1.00	
**When prefer to have children**	Soon(< 1 year)	26(15.9%)	9(5.5%)	2.42(1.07-5.47)	0.033*
	Within 2 years	23(14%)	25(15.2%)	0.77(.40-1.46)	0.428
	After 2 years	93(56.7%)	78(47.6%)	1.00	
**Husband want children in the future**	Yes	142(86.6%)	117(71.3%)	4.24(.86-20.83)	0.075*
	No	20(12.2%)	40(24.4%)	1.75(.33-9.20)	0.509
	Don’t know	2(1.2%)	7(4.3%)	1.00	

**Contraceptive use related characteristics of married women:** over 99% of cases (99.4%) and all (100%) of controls knew at least a method of contraceptive. Injectable was the most known method of family planning among both cases (96.3%) and controls (94.5%), followed by implants (86.6%) for cases and (93.3%) for controls. Withdrawal method was the least known method among the cases (6.1%) and controls (6.1%) (Table 3). Health professionals were the main source of family planning information in the district for both cases (88.4%) and controls (97.6%), followed by neighbors, friends and relatives (53.7%) for cases and 57.9% for controls and then by radio (42.7%) for cases and 40.2% for controls. For both cases and controls, newspaper sources and posters contributed the least to family planning information in the district. Concerning ever use of family planning in their lifetime, the majority of cases (70.1%) used injectables followed by oral contraceptive pills (32.3%). The majority of controls used implants (68.3%) followed by injectables (51.2%). Currently, over three quarter (75.6%) of cases or their partner and 92.1% of controls or their partner were using at least a family planning method. The dominant family planning method in the district were injectables (61.6%) for cases and implants (63.4%) for controls. For those study subjects who were not using contraception, the main reason noted was to have more children, 13.4% of cases and 2.4% of controls. Fear of side effects was cited next by 3.0% of cases and 1.8% of controls. Little pregnancy risk and husband disapproval were the least suggested reasons for not using family planning method from both cases and controls. To plan child spacing, 98 (59.8%) of cases and controls use family planning and 15.2% of cases and 32.3% of controls use family planning for limiting their number of children. Nearly ninety (89.0%) of controls and 67.7% of cases discussed LAPCMs at least once with their husband. Concerning decision to use family planning, 38(23.2%) of cases and 7(4.3%) of controls; a decision is made by their husband. Thirty-eight (23.2%) of cases and 61(37.2%) of controls started family planning, while family planning campaign was undergone in the district. About half (48.8%) of cases and 81.1% of control's husband approved their use of family planning whereas one third (35.4%) of cases and 14.0% of controls opposed. Ninety-six (58.5%) of cases and 142(86.6%) of controls' husband knew whether their partners are using family planning. Forty-seven (28.7%) of cases and 48(29.3%) controls wanted to get from a hospital and 108(65.9%) cases and 109(66.5%) of controls wanted from the health center. Majority of cases (64.0%) and controls (81.7%) discussed with health professionals more frequently and 11.0% - cases and 8.5% - controls discuss at least twice and 5.5% - cases and 6.7% - controls discussed with health professionals at least once in their lifetime about LAPCMs. More than sixty-eight (68.3%) of cases and 90.2% of controls were told what to do if they develop side effects while they are using family planning methods. One hundred and one (61.6%) of cases and 88.4% of controls were told exactly which method to use. Nearly half (48.2%) of cases and one third (34.8%) of controls started using contraceptive methods without informed choice. Health professionals were the most reported to pressurize/persuade respondents to start using contraceptives without informed choice for 47.0% of cases and 32.3% controls.

**Table 3:** Contraceptive use related factors associated with married women’s non-utilization of LAPCMs in bivariable analysis, Chencha district, Southern Ethiopia, 2016

Variables		Cases (n=164)	Controls (n=164)	COR(95%CI)	p-value
**Information about LAPCMs**	Yes	150(91.5%)	158(96.3%)	0.40(.15-1.08)	0.073
	No	14(8.5%)	6(3.7%)	1.00	
**Heard about**	Implant	154(93.9%)	161(98.2%)	3.48(.94-12.9)	0.089
	IUCD	132(80.5%)	139(84.8%)	1.34(.75-2.39)	0.382
	Tubal ligation	40(24.4%)	49(29.9%)	1.32(.81-2.15)	0.321
	Vasectomy	28(17.1%)	21(12.8%)	1.00	
**Reason for not using FP currently**	To have more children	22(13.4%)	4(2.4%)	0.66(.07-5.87)	0.715
	Fear of side effects	8(4.8%)	4(2.4%)	0.26(.02-3.02)	0.286
	Husband disproval	3(1.8%)	0	2.2(.31-15.54)	0.429
	Little pregnancy risk	2(1.2%)	3(1.8%)	0	0.792
	Others	5(3%)	2(1.2%)	1.00	
**Discussion with husband**	Yes	111(67.7%)	146(89%)	0.25(.14-.47)	0.001*
	No	50(30.5%)	17(10.4%)	1.00	
**Husband attitude to LAPCMs**	Approves	80(48.8%)	133(81.1%)	0.18(.07-.44)	0.571
	Opposes	57(34.8%)	23(14%)	0.75(.28-1.99)	0.005
	Don’t know	23(14%)	7(4.3%)	1.00	
**Main decider about LAPCMs**	Both decide together	76(46.3%)	113(68.9%)	1.29(.62-2.68)	0.001
	Husband	38(23.2%)	8(4.9%)	9.37(3.40-25.8)	
	Self	32(19.5%)	17(10.4%)	3.62(.48-8.83)	0.490
	Health profession	13(7.9%)	25(15.2%)	1.00	
**Not based on informed choice**	Yes	79(48.2%)	57(34.8%)	1.81(1.16-2.83)	0.009*
	No	81(49.4%)	106(64.6%)	1.00	
**Who persuade you**	Health professional	77(47%)	53(32.3%)	0.68(.09-5.04)	0.713
	Husband	2(1.2%)	2(1.2%)	1.37(.12-15.5)	0.796
	Others	2(1.2%)	1(0.6%)	1.00	
**Start using Contraceptive methods during a campaign**	Yes	38(23.2%)	61(37.2%)	.52(.32-.85)	0.009*
	No	121(73.8%)	102(62.2%)	1.00	
**Discussion with health professionals**	Yes	131(79.9%)	157(95.7%)	0.15(.06-.37)	0.001*
	No	30(18.3%)	6(3.7%)	1.00	

Note: variables with an asterisk (*) are the candidate for the multivariable model (P-≤0.2)

All variables that were significantly associated with non-utilization of LAPCMs at 5% level of significance by bivariate analysis, were retained for multivariable analysis. The backward stepwise regression was employed and after adjustment, seven variables remained significantly associated with not utilizing LAPCMs. Married women whose partners' educational status is the primary school is significantly more likely not utilize LAPCMs (AOR = 3.1, 95%CI 1.4-6.50; p = 0.003) compared to college and university education. Those married women who have less than or equal to 4 number of alive children are 2.4 (95% CI 1.04-5.67; p = 0.04) times more likely not utilize LAPCMs compared to married women with more than 4 alive children. As depicted in Table 4, the odds of preference to have children in the future for cases was 2.2(95% CI 1.03-4.69; p = 0.042) times greater than the odds for controls. Married women whose partner approves the use of LAPCMs were 0.12 (95% CI 0.03-0.048; p = 0.002) times less likely to not utilize LAPCMs compared to those married women who didn't know their partner's attitude. The odds of husband approval to LAPCMs use for cases were 88% smaller than the odds for the same expose among controls. The other variable which had a significant association with a non-utilization of LAPCMs was informed choice to use contraceptive methods. Accordingly, married women who use contraceptive methods without informed choice were 1.81(95% CI 1.16-2.83; p = 0.006) times more likely not to utilize LAPCMs than those who were informed to use contraceptive methods. Furthermore, married women who started using contraceptive methods during the campaign were 0.52 (95% CI 0.32-0.85; p = 0.021) times less likely not utilizing LAPCMs than those who started contraception use, not through the campaign. The odds of contraceptive use during the campaign for cases were 48% smaller than the odds for the same expose among controls. Discussion with a health professional was found to be 0.31(95% CI 0.10-0.94) times less likely to influence non-utilization of LAPCMs than those who didn't discuss with health professionals (Table 4).

**Table 4:** Predictors of non- utilization of LAPCMs in Chencha district, Southern Ethiopia, 2016

Variables		Cases (n=164)	Control (n=164)	COR(95%CI)	AOR(95%CI)	P-value
**Age**	15-24	17	16	2.80(1.05-7.41)	2.22(.46-10.7)	0.320
	25-29	71	47	3.98(1.81-8.73)	3.75(.94-14.9)	0.061
	30-34	40	35	3.01(1.31-6.90)	2.85(.74-10.8)	0.126
	35-39	25	37	1.78(.75-4.20)	1.39(.37-5.10)	0.618
	40-49	11	29	1.00		
**Educational level of partner**	Can’t read and write	31	39	1.49(.76-2.94)	1.34(.52-3.45)	0.544
	Primary school	62	38	3.06(1.63-5.76)	3.10(1.4-6.50)	0.003*
	Secondary and Preparatory	46	40	2.16(1.13-4.11)	1.83(.86-3.89)	0.111
	College and University	25	47	1.00		
**Number of live children**	≤ 4	133	114	0.82(.73-.92)	2.4(1.04-5.67)	0.040*
	>4	27	50	1.00		
**Like to have children in the future**	Yes	142	111	3.08(1.76-5.37)	2.20(1.03-4.69)	0.042*
	No	22	53	1.00		
**Discussion with husband**	Yes	111	146	0.25(.14-.47)	0.85(.27-2.66)	0.786
	No	50	17	1.00		
**Husband attitude to LAPCMs**	Approves	80	133	0.18(.07-.44)	0.12(.03-.48)	0.002*
	Opposes	57	23	0.75(.28-1.99)	0.45(.13-1.56)	
	Don’t know	23	7	1.00		0.213
**Not informed choice to use contraceptives** **Start using**	Yes	79	57	1.81(1.16-2.83)	2.47(1.30-4.71)	0.006*
	No	81	106	1.00		
**Contraceptives during the campaign**	Yes	38	61	0.52(.32-.85)	0.45(.23-.88)	0.021*
	No	121	102	1.00		
**Discussion with health professionals**	Yes	131	157	0.15(.06-.37)	0.31(.10-.94)	0.039*
	No	30	6	1.00		

Note: variables with an asterisk (*) are statistically significant at p ≤ 0.05

## Discussion

Our study identified sociodemographic, reproductive health history and contraceptive use related factors associated with non-utilization of LAPCMs among married women in Chencha district, Southern Ethiopia. The first factor was related to the educational level of married women's partners. Both women's and their husband's educational status were the significant factors that affect the utilization of LAPCMS [10,12]. In this study, a significant association was observed with partners' educational level. Unlike partners who attained college and university level education, those women whose partners with primary level education were more likely to not utilize LAPCMS (AOR=3.1, 95%CI 1.4-6.50; p=0.003). Our finding is comparable with a study that was conducted in Wolayita town, Southern Ethiopia, women who attained a higher level of education were 2.8 times more intended to utilize LAPCMs than women with no education [8]. The health seeking behaviors like contraceptive methods use by a certain community is strongly affected by their cultural and religious backgrounds [5,13]. Despite this, with regard to religion and ethnicity of married women in this study, there was no significant difference in utilization of LAPCMs. In contrast to the current study, a study from Kenya reported, compared to another religious background such as Muslims and Protestant, the chance of a woman using family planning services is 28 percent lower if she is a Catholic. The difference observed can be due to most of our study subjects were Gamo by ethnicity and Orthodox and Protestant in religion and this might make the association unstable.

The next was related to reproductive health history of married women. Those married women who have less than or equal to 4 number of alive children are 2.4 (95% CI 1.04-5.67; p = 0.04) times more likely not to utilize LAPCMs when compared to married women with more than 4 alive children. Similar to our study, a consistent finding was reported from Adigrat town, northern Ethiopia. Women's intention to use LAPCMs decreases as the number of children the women desired increases [10]. Similarly, the study also revealed that the odds of preference to have children in the future for cases was 2.2(95% CI 1.03-4.69; p = 0.042) times greater than the odds for controls. The observed difference can be due to women's perception of delayed return of fertility after the use of LAPCMs. Moreover, different contraceptive use related factors were related with non-utilization of LAPCMs. Discussion with health providers on family planning use was not associated with non-utilization of LAPCMs. It was revealed that women who discussed with health professionals about LAPCMs had significantly lower odds of not utilizing LAPCMs compared to women who did not discuss (AOR = 0.31, 95% C.I 0.10- 0.94). This might be from understanding the importance of LAPCMs use for ensuring safe and effective protection from unintended pregnancy and also to prevent maternal and newborn deaths. The result of this study showed that partner's disapproval has a strong relation to non-utilization of LAPCMS. The odds of husband approval to LAPCMs use for cases were 88% smaller than the odds for the same expose among controls. A consistent finding was reported from two other studies which were done in Nigeria and Ghana [3,14]. Contraceptive use encourages promiscuity or it affects partner's authority as head of the household was the reported reasons from these two studies. Married women who were not informed to use contraception were more than twice (AOR = 2.47, 1.30-4.71) likely to not utilize LAPCMs than those who were informed about LAPCMs. This finding is almost similar to the report from the Ethiopian Demographic and Health Survey (EDHS) i.e. less than half of (46%) current users of contraceptive methods were informed about the method they used [5]. This study revealed that about 23.2% of married women who were not ever user of LAPCMs and 37.2% ever users of LAPCMs started contraceptive use while a campaign is undergone in their district. A significant difference was observed between the two groups regarding initiation of contraceptive during a campaign. The odds of contraceptive use during a campaign for cases were 48% smaller than the odds for the same expose among controls. This shows the significantly large number of LAPCMs users started the methods during a campaign.

## Conclusion

The result of this study indicates that knowledge about LAPCMs, especially for long-acting reversible contraceptive methods (LARC) is high, and health professionals are the main source of family planning information. Despite this, factors such as educational level of partner, number of live children, future children preference, husband approval to use contraception, not informed choice to contraception use, starting contraceptive methods during campaign and discussion with health professionals about LAPCMs were the most contributing factors for non-utilization of LAPCMs in the district. In order to enhance the utilization of LAPCMs as a bold step towards the improvement of reproductive service utilization and subsequently decreasing maternal and child mortality we recommend first, by educating and motivating the public in the district, concerted efforts should be made to increase the utilization of LAPCMs, especially long-acting permanent contraceptive methods. Second, policies directed towards improving LAPCMs utilization need to consider raising the levels of formal education. Third, training of service providers in quality care, especially about counseling about family planning, the rights of clients, informed the choice of contraceptive methods is very crucial. Different studies reported that expansion of contraceptive methods through campaign has its own drawbacks. Among these, improper counseling, problems related to informed decision making, not following proper procedures and so on. Similarly, there were many married women who started using LAPCMs during a campaign in the district. Thus, a due emphasis should be given to women-friendly services. Finally, different stakeholders in the district, such as women affair, health extension workers, and social ritual groups should give emphasis on couple's knowledge, approval and use of family planning.

### What is known about this topic

- In many developing countries like Ethiopia, access and the utilization of LAPCMs is very low and it also difficult to find them from many reproductive health programs;
- Barriers to the use of LAPCMs are multifactorial and exist at the patient, provider, health system, and national levels;
- Most of the existing evidences about factors related to the non-utilization of LAPCMs are conducted using a cross sectional study design and their findings seem to vary across populations.

### What this study adds

- Provides information on factors associated with the non-utilization of LAPCMs using a strong study design, which can confirm the causal effect relationships of different factors;
- The study was conducted in one of some districts in Ethiopia where unmet need for family planning and birth rate are high. Therefore, the evidence reported from this study could indicate the situation of the problem in the rural settings.

## Competing interests

The authors declare no competing interests.

## References

[1] World Health Organization, UNICEF, United Nations Population Fund, World Bank (2010). Trends in maternal mortality 1990 to 2008: estimates [Internet].

[2] Federal Democratic Republic of Ethiopia Ministry of Health (2010). Health Sector Development Programme IV final draft.

[3] Reference Bureau P (2009). World Population Highlights: Key Findings From PRB’s. World Population Data Sheet [Internet].

[4] World Health Organization (2012). Brown, Global health Europe, WHO joins a call for renewed focus on Family Planning.

[5] Central Statistical Agency [Ethiopia] and ICF International (2017). Ethiopia Demographic and Health Survey 2016.

[6] Mekonnen W, Worku A (2011). Determinants of low family planning use and high unmet need in Butajira District, South Central Ethiopia. Reprod Health.

[7] Alemayehu M, Belachew T, Tilahun T (2012). Factors associated with utilization of long acting and permanent contraceptive methods among married women of reproductive age in Mekelle town, Tigray region, north Ethiopia. BMC Pregnancy Childbirth.

[8] Meskele M, Mekonnen W (2014). Factors affecting women’s intention to use long acting and permanent contraceptive methods in Wolaita Zone, Southern Ethiopia: A cross-sectional study. BMC Womens Health.

[9] Gebremichael H (2014). Acceptance of Long Acting Contraceptive Methods and Associated Factors among Women in Mekelle City, Northern Ethiopia. Sci J Public Heal.

[10] Gebremariam A, Addissie A (2014). Intention to use long acting and permanent contraceptive methods and factors affecting it among married women in Adigrat town, Tigray, Northern Ethiopia. Reprod Health.

[11] (2012). 2007 POPULATION and HOUSING CENSUS OF ETHIOPIA. ADMINISTRATIVE REPORT.

[12] Eliason S, Awoonor-Williams JK, Eliason C, Novignon J, Nonvignon J, Aikins M (2014). Determinants of modern family planning use among women of reproductive age in the Nkwanta district of Ghana: A case-control study. Reprod Health.

[13] Okech TC, Wawire NW, Mburu TK (2011). Contraceptive Use among Women of Reproductive Age in Kenya’s City Slums [Internet]. International Journal of Business and Social Science.

[14] Ghana Statistical Service (1999). Ghana Demographic and Health Survey 1998, Accra, Ghana, and Claverton, USA.

